# Investigation into the *in vivo* mechanism of diosmetin in patients with breast cancer and COVID-19 using bioinformatics

**DOI:** 10.3389/fphar.2022.983821

**Published:** 2022-08-17

**Authors:** Jin Wang, Shanbo Ma, Long Li, Yuhan Chen, Qian Yang, Feiyan Wang, Meiling Zheng, Shan Miao, Xiaopeng Shi

**Affiliations:** ^1^ Department of Pharmacy, Xijing Hospital, Air Force Military Medical University, Xi’an, Shaanxi, China; ^2^ School of Pharmacy, Shaanxi University of Traditional Chinese Medicine, Xianyang, Shaanxi, China

**Keywords:** breast cancer, COVID-19, Diosmetin, computational biology, prognosis

## Abstract

Patients with breast cancer are prone to SARS-CoV-2 infection [the causative virus of coronavirus disease (COVID-19)] due to their lack of immunity. In the current study, we examined the mechanism of action of Diosmetin, a flavonoid with anti-inflammatory properties, in patients with BRCA infected with SARS-CoV-2.We used bioinformatics technology to analyze the binding ability, biological function, and other biological characteristics of Diosmetin *in vivo* and examine the core target and potential mechanism of action of Diosmetin in patients with patients with breast cancer infected with SARS-CoV-2. A prognostic model of SARS-COV-2–infected breast cancer patients was constructed, and the core genes were screened out, revealing the correlation between these core genes and clinicopathological characteristics, survival rate, and high-risk and low-risk populations. The docking results revealed that Diosmetin binds well to the core genes of patients with breast cancer with COVID-19. Gene Ontology (GO) and Kyoto Encyclopedia of Genes and Genomes (KEGG) analyses suggested that Diosmetin inhibited inflammation, enhanced immune function, and regulated the cellular microenvironment in patients with BRCA/COVID-19. For the first time, we reveal the molecular functions and potential targets of Diosmetin in patients with breast cancer infected with SARS-CoV-2, improving the reliability of the new drug and laying the foundation for further research and development.

## Introduction

Although medical science has developed rapidly in the past few decades, infectious diseases continue to pose a serious threat to human life and health. Moreover, the current coronavirus disease (COVID-19) pandemic (To et al., 2021) represents a considerable threat to human life and has resulted in a high proportion of death worldwide. COVID-19, caused by SARS-CoV-2, was first named by the National Health Commission as novel coronavirus pneumonia (Dong et al., 2020), before being termed COVID-19 by the World Health Organization (WHO). The transmission route of the virus is similar to that of influenza, occurring mainly through the respiratory tract (Ahn et al., 2020). Until now, the cumulative number of confirmed COVID-19 cases worldwide has reached nearly 488 million, and the cumulative death toll has reached 6.14 million. Consequently, research on anti-COVID-19 drugs remains a top priority.

According to a WHO report, breast cancer is the second most common cause of cancer-related death in women worldwide. At present, surgical resection, radiotherapy, chemotherapy, endocrine drug therapy, targeted drug therapy, and photodynamic therapy are the common interventions for breast cancer (Montagna et al., 2019; Morganti and Curigliano, 2019). According to numerous studies, cancer occurrence, growth, and metastasis are all closely associated with chronic inflammation and cancer may even be classified as an inflammatory chronic condition (Zhao et al., 2022). During the COVID-19 pandemic, most hospitalized patients are at increased risk of infection due to the pathogenic characteristics of COVID-19. In line with this, it is difficult to treat patients with BRCA infected with SARS-CoV-2 due to their low immunity and the lack of effective anti-COVID-19 drugs (Breast Cancer Expert Committee of National Cancer Quality Control Center, 2022). Therefore, it is necessary to evaluate the effectiveness of drugs for such patients.

Diosmetin is a natural flavonoid compound that is present in legumes, olive leaves, and citrus plants and has various biological activities (Meephat et al., 2021), such as antioxidant, anti-infective, anti-tumor, anti-mutagenic, and anti-allergic properties, as well as positive effects in neurological and ophthalmic diseases. Diosmetin also has cytotoxic effects in various tumors, including breast cancer and nasopharyngeal carcinoma (Zhao et al., 2021). As an effective antioxidant, the protective effect of Diosmetin on some harmful oxidation-induced reactions has attracted extensive attention, especially in the fields of biology, medicine, nutrition (Ma and Zhang, 2020; Luo et al., 2021). Moreover, several *in vitro* and *in vivo* studies have found that Diosmetin has good protective effects on liver, lung, and kidney damage (Lee H. et al., 2020; Wang W. et al., 2020).

**FIGURE 1:**
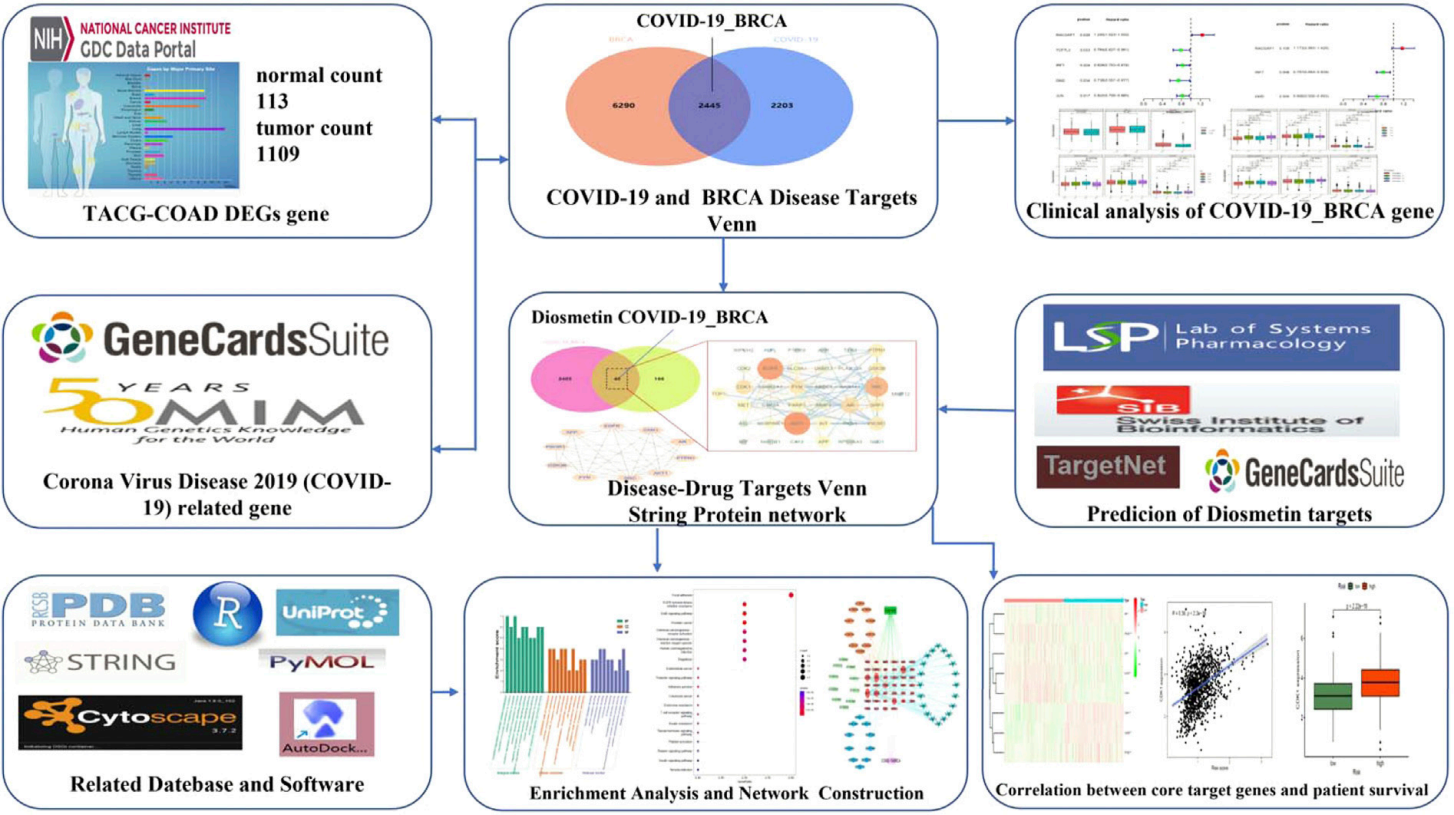
Study workflow. Computational bioinformatics analysis technique; the figure depicts the action and mechanism of Diosmetin against BRCA/COVID-19.

Bioinformatics is concerned with collecting, processing, storing, disseminating, and annotating biological data. As a new discipline (Dinov, 2016), it uses computer science and biology to unravel biological mysteries posed by enormous and complicated life data (Fernandez-Pozo et al., 2022). Genomics and proteomics are the key modules for research; specifically, nucleic acid and protein sequences are analyzed to establish the biological information of the structure and function (Zhang et al., 2022). In this study, the *in vivo* mechanism of Diosmetin on breast cancer patients infected with COVID-19 was explored by means of bioinformatics, network pharmacology, and molecular docking. Figure 1 is the flowchart of this study.

## Materials and methods

### Screening of breast cancer/COVID-19-related genes

The transcriptome genes of patients with breast cancer were retrieved from The Cancer Genome Atlas (TCGA: www.cancer.gov/about-cancer) to determine genes linked to COVID-19. We used the “limma” package of the R language Bioconductor to screen and evaluate the differential genes of patients with breast cancer, with *p* < 0.05 and log(FC) > 1. GeneCards and the OMIM database were searched for COVID-19-related genes (Amberger et al., 2019), and the differential genes of breast cancer were intersected with COVID-19-related genes to obtain overlapping targets of BRCA/COVID-19 (Stelzer et al., 2009).

### Clinicopathological study of breast cancer/COVID-19 core genes

The connection between genes associated with patients with breast cancer and COVID-19 and patient survival was investigated using the R language “survival” package. Univariate Cox proportional hazards regression analysis was used to conduct prognostic analysis (Wang L. et al., 2020). The related clinicopathological characteristics of the signature genes and patients with breast cancer infected with SARS-CoV-2 were analyzed using a multivariate Cox proportional hazards regression model. Finally, patients were classified into low- and high-risk categories based on the median risk score (Li Z. et al., 2021).

### Pharmacological target acquisition of Diosmetin

The pharmacological targets of Diosmetin were investigated and collected using web databases, including TCMSP, Swiss Target Prediction (Daina et al., 2019), TargetNet (Yao et al., 2016), and GeneCards (Stelzer et al., 2016). After data correction through the UniProt database (UniProt Consortium, 2021), duplicate genes were deleted to determine candidate genes.

### Enrichment analysis and network visualization

The R language packages “ClusterProfiler,” “ReactomePA,” “org.Hs.eg.Db,” and “GOplot” were used to intersect patients with breast cancer with COVID-19 with the intersection of Diosmetin target genes. The intersection genes were subjected to GO enrichment analysis and KEGG pathway enrichment analysis and visualization. A Venn diagram was used to obtain the intersection gene targets in BRCA/COVID-19 and Diosmetin, and Cytoscape software (Version 3.7.2) was used to construct a “drug intersection target–Go function–pathway–disease” network map (Chan, 2018).

### Identifying the core target of Diosmetin in breast cancer/COVID-19

The BRCA/COVID-19 gene and Diosmetin intersection genes were put into the STRING database to obtain the core protein–protein interaction (PPI) network diagram, and the file was downloaded in tsv format. The Network-Analyzer component of the Cytoscape software was used to conduct topological analysis. The target network had a median degree of freedom of 7.9 and a maximum degree of freedom of 26. The degree of freedom range for the core gene screening criteria was defined at 4–26. The degree value was used to collect core targets. The R language was used to visualize the correlation between core genes and the survival rate of high and low risk groups (Szklarczyk et al., 2021).

### Molecular docking analysis

Molecular docking analysis was used to verify the binding and interaction force between the corresponding protein receptor and the small molecule ligand. The PubChem database was used to elucidate the chemical structure of Diosmetin (Kim et al., 2021), and the Protein Data Bank (PDB) database was used to identify COVID-19-related protein structures. The ChemBio3D Draw module of ChemBio Office software was used to conduct MM2 force field optimization of small molecule structures (version 2010). The protein receptors and small molecule ligands were dehydrated using PyMOL visualization tools and saved as PDB files. MGLTools 1.5.6 from AutoDock Vina program was used to process the relevant protein receptors, hydrogenation, and the Gasteiger charge of merging non-polar hydrogen atoms (Johnson et al., 2015). Finally, the raw pdb file was converted into a PDBQT file format (Trott and Olson, 2010). Adjust the parameters in the Auto-Dock program to define the location of the active docking pocket.

## Results

### Identification of breast cancer with COVID-19 target genes

**FIGURE 2:**
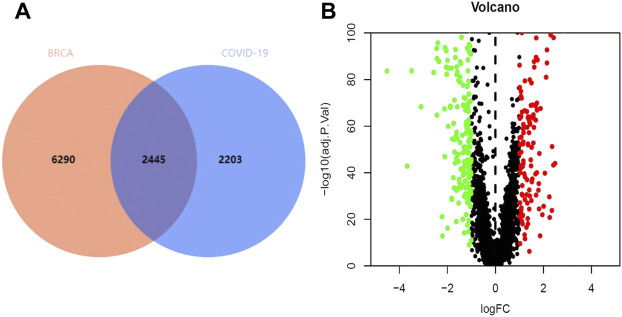
Intersection genes in BRCA/COVID-19. **(A)** Venn diagram of intersecting genes in BRCA/COVID-19. **(B)** Volcano plot of differential gene expression.

Through the GeneCards and OMIM database, 4,648 genes related to COVID-19 were collected, and 8,735 genes were analyzed in TCGA database to identify the genes that were associated with BRCA (Figure 2A). The results revealed that 337 genes were associated with both COVID-19 and BRCA. We also performed a differential analysis of the crossover genes using the R language, and the results revealed that 219 genes were downregulated and 119 were upregulated (Figure 2B).

### Clinicopathological analysis of breast cancer/COVID-19-related genes

**FIGURE 3:**
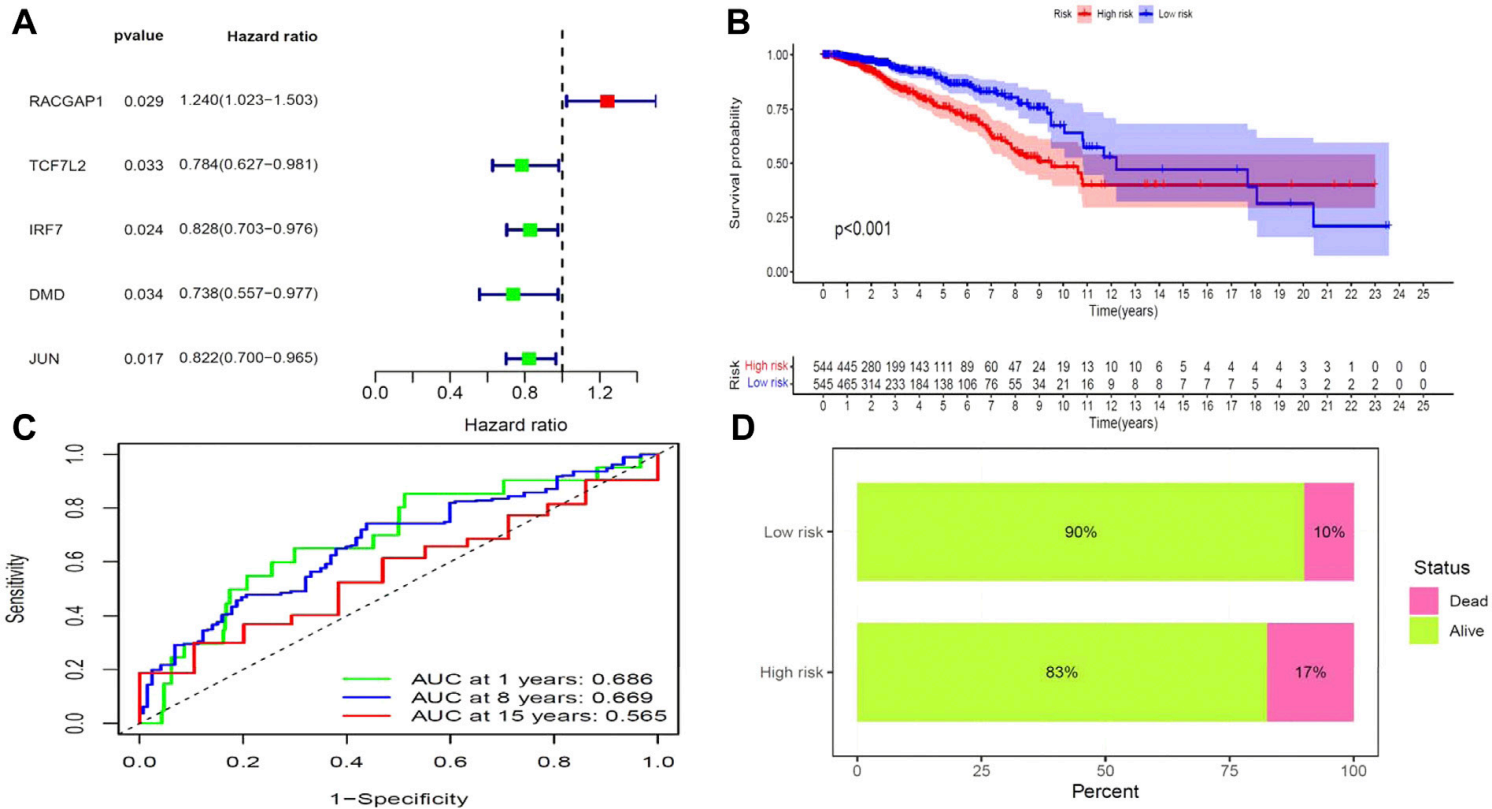
Prognostic value of BRCA/COVID-19-related genes. **(A)** Univariate Cox analysis identified five genes, including *RACGAP1*, *TCF7L2*, *IRF7*, *DMD*, and *JUN*. **(B)** Survival analysis showed a difference in overall survival between high- and low-risk groups. **(C)** Accuracy of the receiver operating characteristic (ROC) curve in predicting the prognostic survival rate. **(D)** The survival rate of patients in the low-risk group was significantly higher than that in the high-risk group.

**FIGURE 4:**
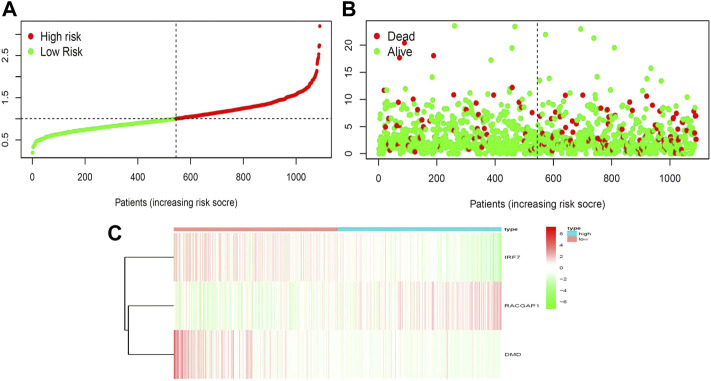
Correlation between survival and risk score. **(A)** Analysis of patients’ risk score using Cox proportional hazards regression. **(B)** Surviving and dying patient risk score results. **(C)** Heatmap of core gene expression levels.

**FIGURE 5:**
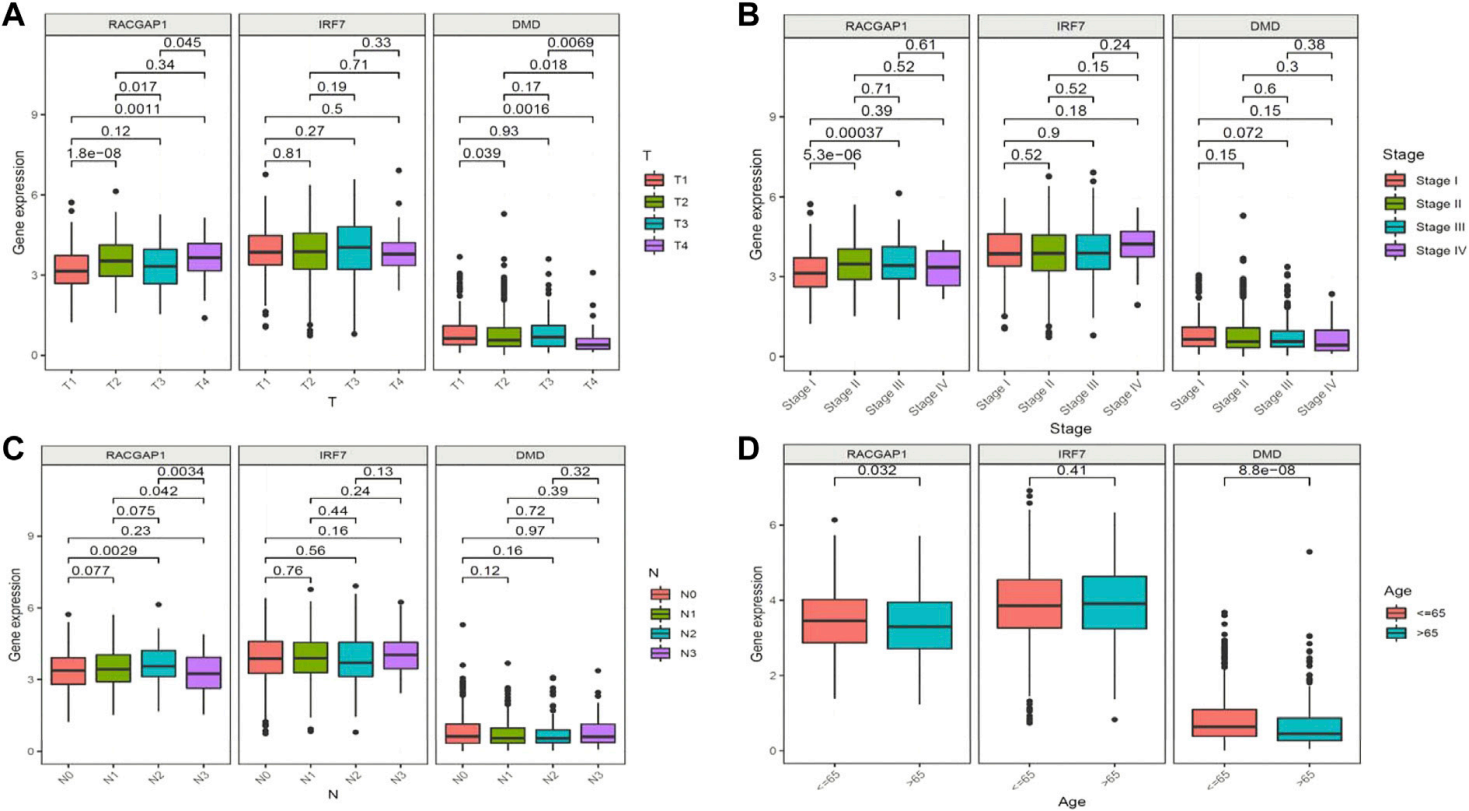
Clinical prognostic model and gene correlation analysis. **(A)** Association of the expression of *RACGAP1*, *IRF7*, and *DMD* with tumor stage and metastasis in patients with breast cancer (BRCA). **(B)** Association of the expression of *RACGAP1*, *IRF7*, and *DMD* with the number of lymph node metastases. **(C)** Association of the expression of *RACGAP1* with BRCA staging. **(D)** Association of the expression of *RACGAP1*, *IRF7*, and *DMD* with age.

We next aimed to analyze the association between sensitive genes and COVID-19/BRCA disease. The results of Cox analysis revealed that the RACGAP1, TCF7L2, IRF7, DMD, and JUN were significantly associated with the development of COVID-19/BRCA (*p* < 0.05) (Figure 3A; Table 1). The patients were then categorized into high- and low-risk groups based on their risk score. In the overall survival analysis, Significant difference between high and low risk groups (Figure 3B). The results of the area under the receiver operating characteristic curve (ROC) indicated that the model was relatively reliable in predicting patient survival at 1 and 8 years (Figure 3C). The histogram shows that the low-risk group had a significantly higher survival rate than the high-risk group (Figure 3D). The survival status, risk score, and expression distribution of the above five genes were analyzed for each patient with BRCA, and the results showed that the higher the patient’s risk value, the higher the risk score (Figure 4). We next performed independent univariate and multivariate prognostic analyses for these five genes. The clinical correlation pathological study of RACGAP1, IRF7, and DMD showed that RACGAP1 was highly expressed in the high-risk group, indicating that RACGAP1 has a greater impact on patients with breast cancer infected with SARS-CoV-2 (Figure 4C; Table 2). RACGAP1 also showed significant differences according to the T stage (Figure 5A), with lower expression in T1 stage and higher expression in T2 to T4. This result demonstrated that the expression of RACGAP1 gradually increased with the progression of the disease. The expression of RACGAP1 was also related to the number and extent of lymph node metastasis and the spread in the lymph node region. RACGAP1 did not metastasize in the N0 stage, while a portion of the patient began to metastasize in the N1 stage (Figure 5C). Moreover, the expression of RACGAP1 in stage II, stage III, and stage IV was higher than that in stage I (Figure 5B). In terms of age, the expression of RACGAP1 in the population aged ≤65 years was greater than that in the population >65 years (Figure 5D). The risk histogram demonstrates that the mortality rate of patients in the low-risk group was significantly lower than that in the high-risk group (Figure 3D).

**TABLE 1:** Univariate Cox proportional hazards regression analysis of COVID-19 combined with BRCA gene.

Symbol	HR	HR.95L	HR.95H	*p*-value
RACGAP1	1.23986	1.02252	1.50341	0.02878
TCF7L2	0.78441	0.62724	0.98097	0.03331
IRF7	0.82838	0.70338	0.97559	0.024062
DMD	0.73763	0.55672	0.97733	0.034033
JUN	0.82214	0.70025	0.96525	0.01676

HR, hazard ratio.

HR.95L and HR.95H: risk fluctuation ranges.

**TABLE 2:** Multivariate Cox proportional hazards regression analysis.

Symbol	Correlation coefficient	HR	HR.95L	HR.95H	*p*-value
RACGAP1	0.159806	1.173283	0.965353	1.426	0.108347
IRF7	−0.23991	0.786703	0.662691	0.933921	0.006124
DMD	−0.40275	0.668482	0.500452	0.892929	0.006398

### Identification of the intersection genes of Diosmetin and COVID-19/breast cancer

**FIGURE 6:**
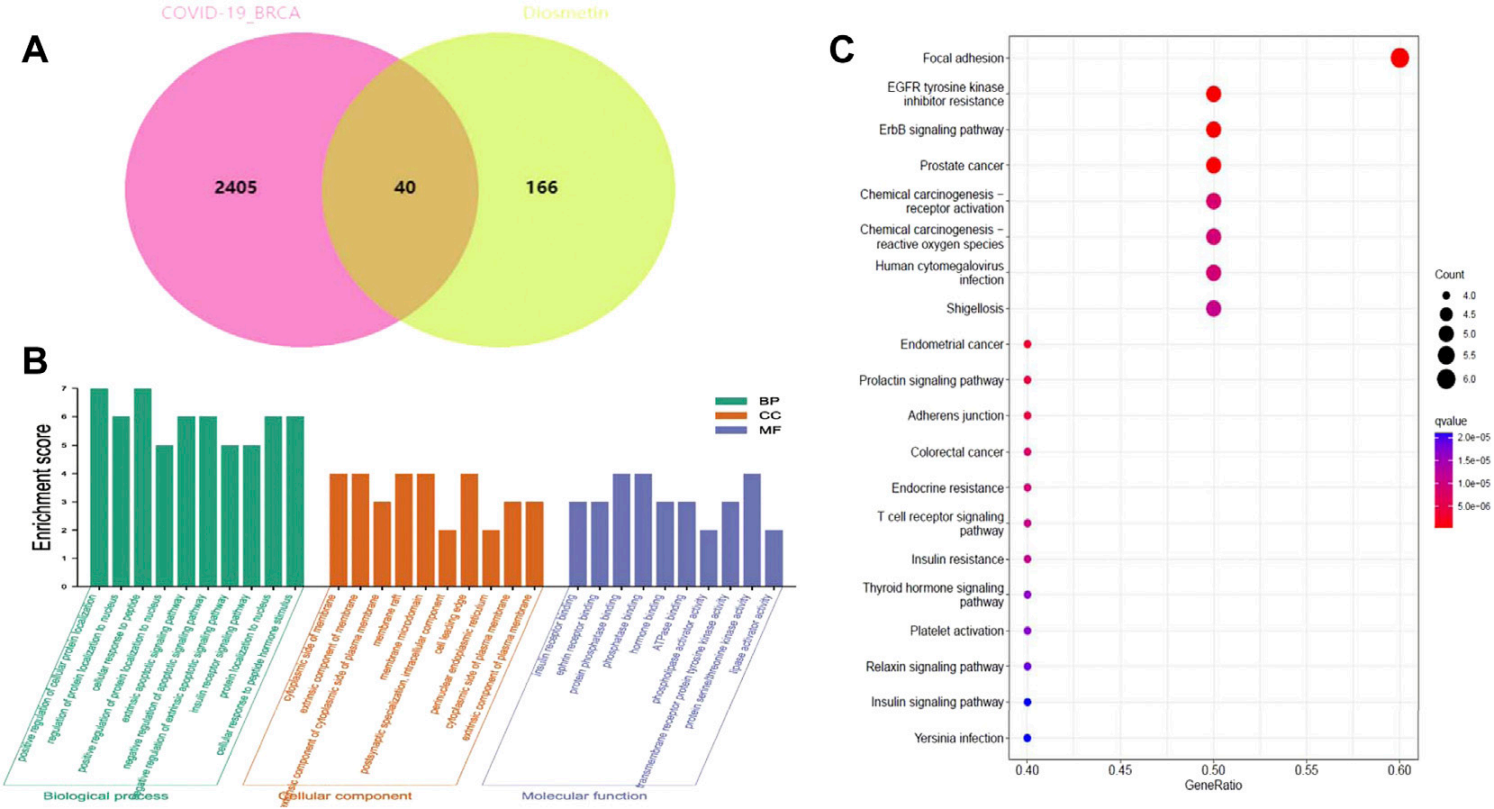
Functional identification of BRCA/COVID-19 crossover genes by Diosmetin. **(A)** Venn diagram of the intersection of Diosmetin and BRCA/COVID-19 genes. **(B)** Gene Ontology analysis of Diosmetin and BRCA/COVID-19 intersecting genes. **(C)** Kyoto Encyclopedia of Genes and Genomes (KEGG) pathway for Diosmetin and BRCA/COVID-19 gene crossover.

**FIGURE 7:**
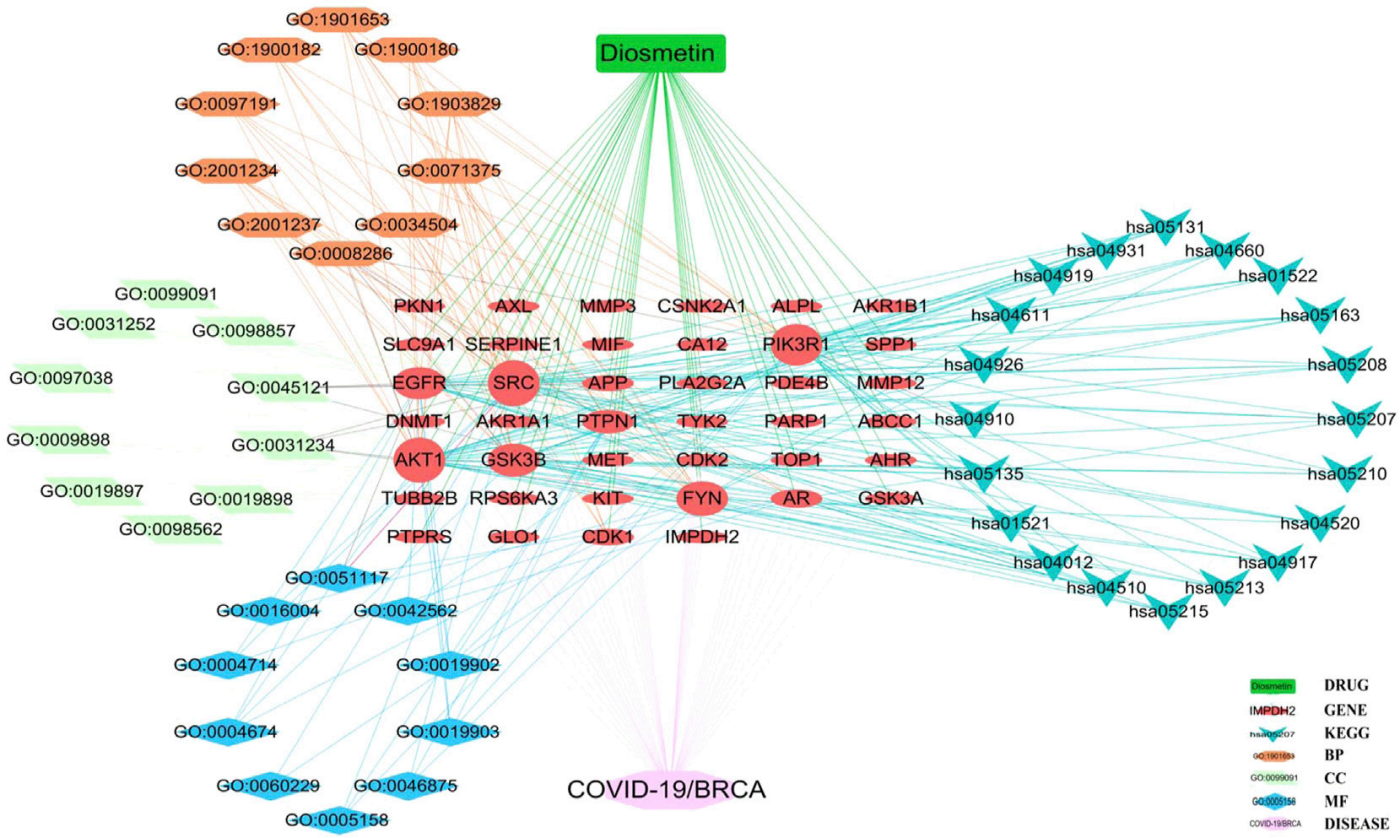
Core biological targets, pharmacological functions, and interaction networks of the signaling pathways of Diosmetin against BRCA/COVID-19.

The pharmacological targets of Diosmetin were investigated and collected using the TCMSP, Swiss Target Prediction (21), TargetNet (22), and GeneCards (23) web databases. After data correction through the UniProt database (24), duplicate genes were deleted to determine candidate genes. In addition, 40 intersecting genes were identified by the intersection of COVID-19/BRCA intersection genes and related targets of Diosmetin (Figure 6A). GO enrichment analyses of the 40 intersecting genes showed that Diosmetin affects a series of *in vivo* processes, including positive regulation of cellular protein localization, regulation of nuclear localization, cellular response to peptides, positive protein localization regulation of the nucleus, extrinsic apoptosis signaling pathway, negative regulation of the apoptosis signaling pathway, negative regulation of the extrinsic apoptosis signaling pathway, insulin receptor signaling pathway, protein localization in the nucleus, cellular response to peptide hormone stimulation, insulin receptor binding, ephrin receptor binding, protein phosphatase binding, phosphatase binding, hormone binding, ATPase binding, phospholipase activator activity, transmembrane receptor protein tyrosine kinase activity, protein serine/threonine kinase activity, and lipase activator activity (Figure 6B). Additionally, in KEGG pathway enrichment analysis, 20 KEGG pathways were found to be associated with all core targets (P-adjust < 0.05); these included EGFR tyrosine kinase inhibitor resistance, ErbB signaling, focal adhesions, prostate cancer, endometrial cancer, prolactin signaling, adhesion junctions, colorectal cancer chemo-onco-receptor activation, chemo-oncogenic-activity oxygen, human cytomegalovirus infection, endocrine resistance, T cell receptor signaling, shigellosis, insulin resistance, thyroid hormone signaling, platelet activation, relaxin signaling, insulin signaling, and Yersinia bacterial infection (Figure 6C) (Figure 7).

### Identifying the core target of Diosmetin against COVID-19/breast cancer

**FIGURE 8:**
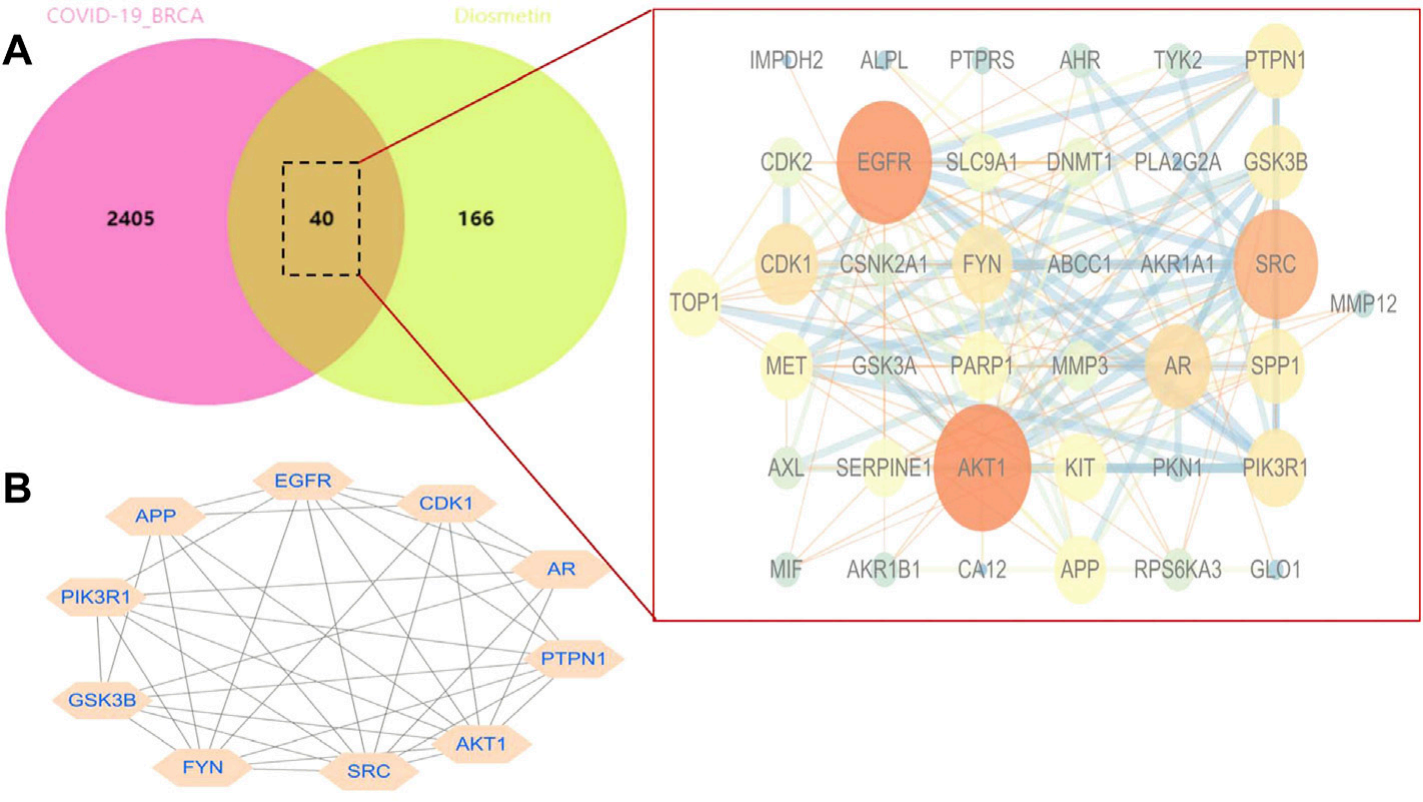
Network analysis of Diosmetin anti-BRCA/COVID-19 genes. **(A)** STRING analysis of the protein–protein interaction network mediated by 40 Diosmetin cross-targets. **(B)** Cellular landscape analysis representing the protein interaction network associated with Diosmetin anti-COVID-19/BRCA effects. The 10 core targets (EGFR, PIK3R1, SRC, AKT1, AR, FYN, PTPN1, APP, CDK1, and GSK3B) are highlighted.

**FIGURE 9:**
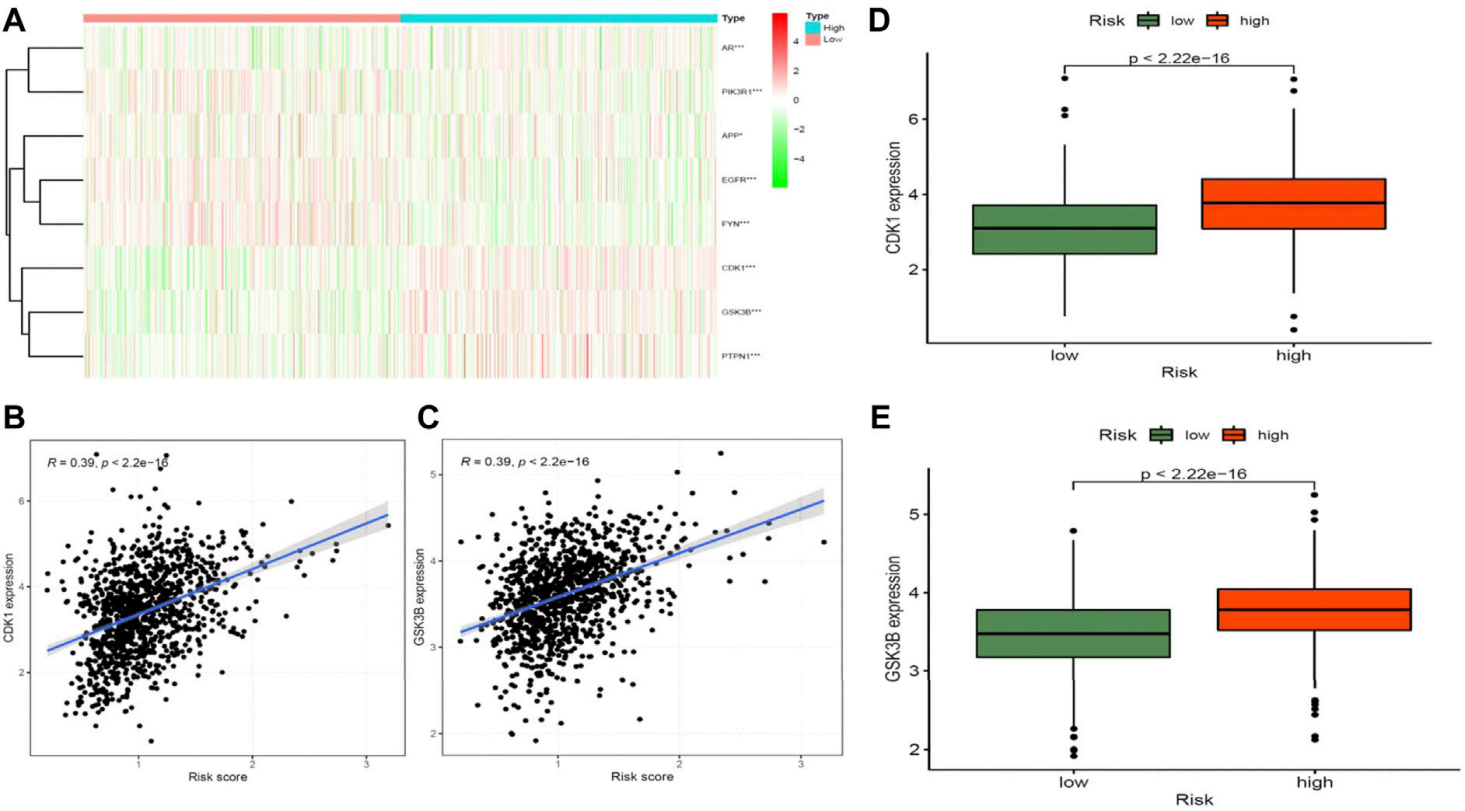
Correlation analysis between core target genes of drugs and diseases and disease risk. **(A)** Heat map of core genes and disease risk groupings. **(B–D)** CDK1 is positively associated with disease risk scores and is highly expressed in the high-risk groups. **(C–E)** GSK3B is positively associated with disease risk scores and is highly expressed in the high-risk groups.

STRING analysis was used to determine the PPI network mediated by the 40 Diosmetin crossover genes against COVID-19/BRCA (Figure 8A). All mapped crossover genes were input into Cytoscape software, and topological parameters were calculated and ranked by degree value. Ten core gene targets were identified by analysis, including *EGFR*, *PIK3R1*, *SRC*, *AKT1*, *AR*, *FYN*, *PTPN1*, *CDK1*, *APP* and *GSK3B* (Figure 8B). The correlation heat map of the core genes showed that PTPN1, CDK1, and GSK3B were highly expressed in the high-risk group (Figure 9A), while CDK1 and GSK3B were positively correlated with risk scores (Figures 9B–E).

### Binding of Diosmetin to potential target genes of COVID-19/breast cancer

**FIGURE 10:**
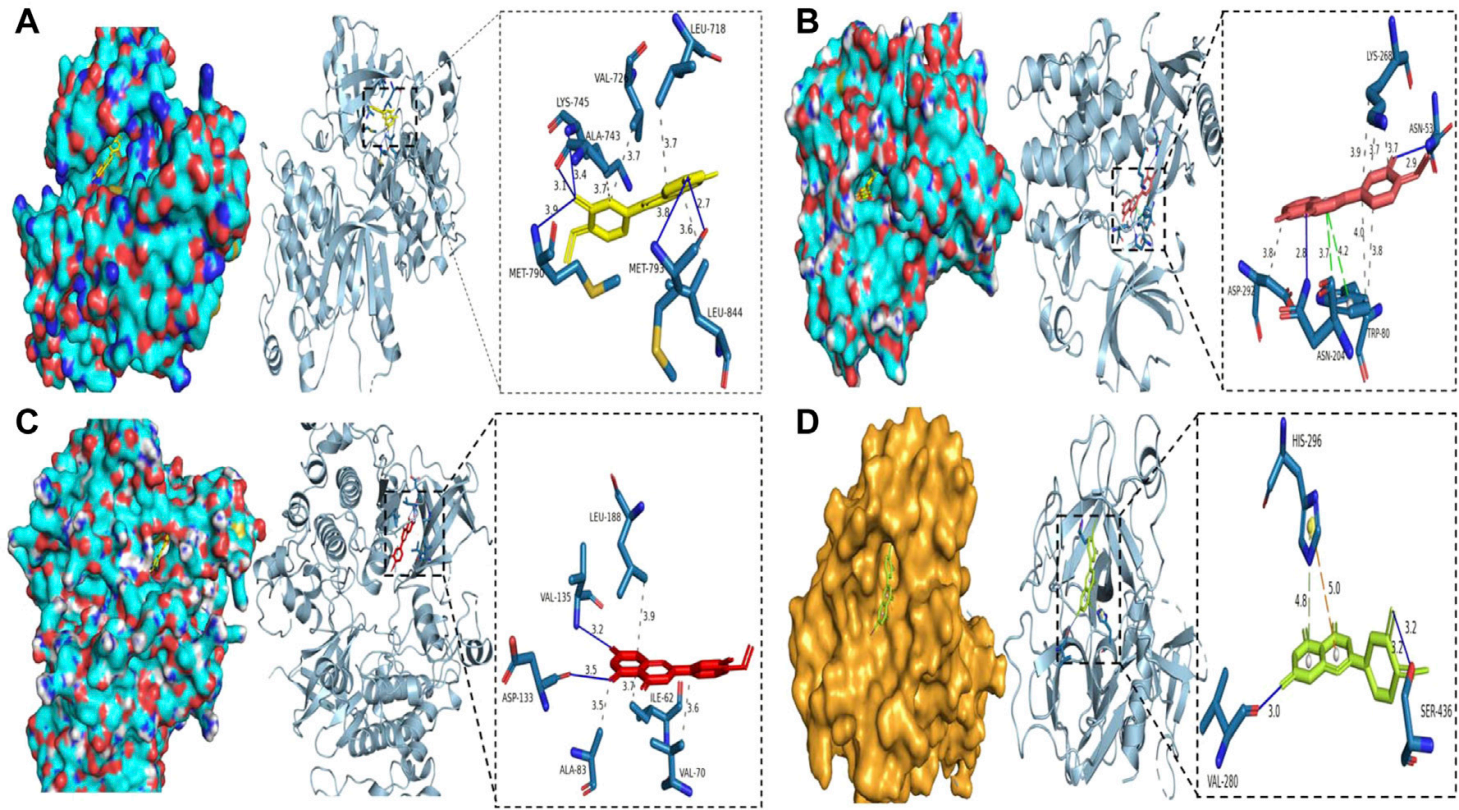
Binding of Diosmetin to COVID-19/BRCA and the core targets EGFR, AKT1, GSK3B using molecular docking analysis. **(A)** Diosmetin and the EGFR protein 6TG0 form hydrogen bonds on LYS-745, MET-790, ALA-743, and MET-793, with hydrophobic bonds on LEU-718, ALA-743, and VAL-726. **(B)** Diosmetin and AKT1 protein 7NH5 form hydrogen bonds on ASN-53 and ASN-204, with hydrophobic bonds on ASP-292, TRP-80, and LYS-268, and *π* bonds on ASN-204 and TRP-80. **(C)** Diosmetin and GSK2B protein 6Y9S form hydrogen bonds on VAl-135 and ASP-133, with hydrophobic bonds on LEU-188, ALA-83, ILE-62, and VAL- 70. **(D)** The Diosmetin and COVID-19 protein 7MEQ forms a hydrogen bond on VAL-280 and SER-436, with a cation interaction on HIS-296 and a *π* bond on HIS-296.

Molecular docking analysis of the COVID-19 core target was performed to confirm the effects of Diosmetin on this protein. The structure of the TMPRSS2 major protein was obtained using the PDB database (Figure 10). The protein receptor activity pocket parameters were set to center x, y, and z of 7.842, −5.136, and 12.172, respectively, and X, Y and Z size of 126. The docking results show that the amino acid residues of small molecules and protein receptors have hydrogen bonds and interactions at VLA-280 (3.0 Å) and SER-436 (3.2 Å), and form cationic interactions at HIS-296 (5.0 Å), with a *π* bond (4.8 Å) formed at HIS-296 (Figure 10D), indicating that Diosmetin has a good binding ability of the TMPRSS2 protein (COVID-19). We also analyzed the possible binding between the small molecule compound geranin and the 10 core targets (EGFR, PIK3R1, SRC, APP, AKT1, AR, FYN, PTPN1, CDK1, GSK3B). The results showed that the docking free energy fractions of EGFR, AKT1, and GSK3B were low, at −9.3 kcal/mol, −9.4 kcal/mol, and −9.3 kcal/mol, respectively, indicating good docking results (Figure 10A, Figure 10B, Figure 10C).

## Discussion

With the continuous variation of SARS-CoV-2, the number of infected individuals worldwide is increasing rapidly every day (Guc et al., 2022; Lee and Morling, 2022; Salmi, 2022). Additionally, the rapid emergence and evolution of cancer over the past few decades have led to the development of various chronic conditions, such as immune dysfunction and immunosuppression, both of which can affect the quality of life of patients (Kumar, 2021). Currently, COVID-19 is widely prevalent, and hospitals are high-risk for exposure to SARS-CoV-2, thereby increasing the risk of infection in patients who are hospitalized with cancer (Liu et al., 2020). According to cancer statistics from 2020, BRCA causes the highest cancer-related deaths worldwide (Siegel et al., 2022). The current treatment guidelines for patients with breast cancer do not prevent infection with SARS-CoV-2, which further reduces the survival rate.

Studies have shown that Diosmetin is cytotoxic to BRCA cells (Androutsopoulos et al., 2009). Based on its anti-inflammatory and antiviral effects (Lee D. H. et al., 2020; Li et al., 2022), we hypothesized that Diosmetin might have an effective pharmacological activity on patients with BRCA/COVID-19. In the previous analysis, we identified 337 core genes that were associated with the development of COVID-19 and were also involved in the development of breast cancer, among which 219 were upregulated and 119 were downregulated. DGE-based analysis can be used to determine the clinical features of patients with breast cancer and COVID-19. Independent prognosis and survival analysis revealed important differential genes, including RACGAP1, TCF7L2, IRF7, DMD, and JUN, which can be used as effective biomarkers for screening and characterizing patients with BRCA and COVID-19 at all stages. Indeed, these 337 crossover genes can be used as potential therapeutic targets for patients with BRCA complicated by COVID-19. Additionally, using network pharmacology, we identified 40 core genes of Diosmetin for BRCA combined with COVID-19. Among these, EGFR, SRC, AKT1, and PIK3R1 showed a high correlation with related proteins. EGFR, via regulating angiogenesis and vascular remodeling and stimulating cell proliferation, promotes inflammation, wound healing, tissue repair, and oocyte maturation (Sigismund et al., 2018), and is considered a main driving factor of tumorigenesis (Sabbah et al., 2020). Although inappropriate activation of EGFR in cancer is mainly caused by amplification and point mutation of genomic sites, transcription upregulation or ligand overproduction are also induced by autocrine/paracrine mechanisms (Wu and Zhang, 2020). EGFR is increasingly considered a biomarker of tumor drug resistance, and its amplification or secondary mutation occurs under the pressure of drugs (Jang et al., 2020). SRC, a proto-oncogene complex protein kinase, promotes the development of cancer in many respects (Li L. et al., 2021). SRC kinase plays an important role in promoting tumor growth and progression, and its activity is related to the low survival rate of patients (Dasgupta et al., 2018). Moreover, steroid receptor coactivator 3 (SRC-3) is a family of SRCs, also known as Breast Cancer Amplification 1 (AIB1) (Okuzaki et al., 2020). AKT1-E17K mutation in breast cancer can isolate β-catenin to the cell membrane, which decreases ZEB1 transcription and leads to an increase in E-cadherin expression and the reversal of epithelial-mesenchymal transformation, thus inhibiting tumor migration and invasion (John et al., 2022). The growth of human solid tumors depends not only on rapidly proliferating cancer cells, but also on the continuous production and stationary proliferation of AKT1 (Alves et al., 2018). Moreover, the activation of the PIK3R1-PI3K pathway contributes to the occurrence and development of tumors (Chen et al., 2018). PIK3R1 somatic cell mutation has a similar mechanism to cancer-related mutation, which leads to complex vascular malformation and overgrowth (Cottrell et al., 2021). Of the 40 core genes that were identified as potential targets of Diosmetin, the anti-cancer effects were mainly controlled by the anti-inflammatory and antiviral properties of the drug. These effects were also influenced by the regulation of various signal pathways, such as Th17 cell differentiation and the NF-kappa B pathway. Based on the above analysis results, the anti-BRCA/COVID-19 effect of Diosmetin may be regulated by core genes, including *EGFR, PIK3R1, SRC*, *AKT1*, *APP*, *AR*, *FYN*, *PTPN1*, *CDK1*, and *GSK3B.* Through further verification of molecular docking, we determined that the small molecule ligands bound well to protein molecule receptors, which indicates that Diosmetin has a certain effect on the 7MEQ structure of TMPRSS2 protein, an important protein of COVID-19. Additionally, the results show that Diosmetin has excellent binding with 6TG0, 7NH5, and 6Y9S in the core target EGFR, AKT1, and GSK3B proteins, respectively. The results show that Diosmetin can highly bind with novel coronavirus-specific proteins and core gene proteins, suggesting that the effect of Diosmetin on BRCA/COVID-19 may be targeted by EGFR, AKT1, and GSK3B proteins.

## Conclusion

Numerous bioinformatics data and calculation results highlight that immunomodulation, antiviral, and anti-inflammation are the key mechanisms of Diosmetin for treating BRCA/COVID-19. Additionally, according to the established pharmacological function and therapeutic mechanism, Diosmetin can be used to treat BRCA/COVID-19. In the current research, we have determined the pharmacological targets of Diosmetin for BRCA/COVID-19, paving the way for further experiments to provide additional clues about the underlying mechanism of action.

## Data Availability

The original contributions presented in the study are included in the article/Supplementary Material, further inquiries can be directed to the corresponding authors. All authors contributed to the article and approved the submitted version

## Abbreviations

BRCA, Breast cancer; COVID-19, Coronavirus disease-2019; SARS-CoV-2, Severe acute respiratory syndrome coronavirus 2; TCGA, The Cancer Genome Atlas; GO, Gene ontology; BP, Biological process; KEGG, Kyoto Encyclopedia of Genes and Genomes; PDB, Protein Data Bank.
